# Transcriptomic changes in the lacrimal glands of a Sjogren’s disease animal model highlight key molecular mediators and altered biological functions underpinning glandular inflammation and hypofunction

**DOI:** 10.3389/fopht.2026.1697924

**Published:** 2026-03-13

**Authors:** Danny Toribio, Junji Morokuma, Albert Tai, Driss Zoukhri

**Affiliations:** 1 Department of Basic and Clinical Translational Sciences, Tufts University School of Dental Medicine, Boston, MA, United States; 2 Department of Immunology, Tufts University School of Medicine, Boston, MA, United States; 3 Department of Ophthalmology, Tufts University School of Medicine, Boston, MA, United States

**Keywords:** autoimmune disease, dry eye disease, inflammation, lacrimal gland, RNA-Seq, Sjogren’s disease, transcriptomic analysis

## Abstract

**Introduction:**

Sjogren’s disease (SjD) is a chronic autoimmune condition that targets the lacrimal glands (LGs), resulting in aqueous-deficient dry eye disease (DED). Insufficient or low-grade tear secretion onto the ocular surface affects the normal physiology of the cornea and conjunctiva and can result in ocular surface damage. Herein, we evaluated the transcriptomic profiles of diseased versus healthy LGs to investigate the underlying molecular mechanisms underscoring LG autoimmune inflammation and secretory hypofunction.

**Methods:**

LGs were removed from male NOR/LtJ mice (SjD mouse model) and sex- and age-matched BALB/c controls at 3 weeks (pre-disease), 8 weeks (early disease onset), and 16 weeks (intermediately advanced disease stage). LGs were processed for either RNA extraction or histopathological staining. Bulk RNA sequencing was conducted for each LG sample followed by downstream data processing for differential expression analysis using the DESeq2 R package. Biological interpretation of the differential gene expression datasets was achieved using the QIAGEN Ingenuity Pathway Analysis (IPA) software. IPA was used to compare the differential gene expression results obtained in our analyses to those of previously published, publicly available datasets from other SjD-related transcriptomic-based studies.

**Results:**

The number of differentially expressed genes between NOR and BALB/c mice increased in correlation with the development and progression of dacryoadenitis. The most significantly upregulated pathways were primarily related to immune responses, emphasizing the activation of cellular and cytokine-mediated inflammatory responses. Several cytokines, transcription factors, and toll-like receptors were significantly predicted to drive most of the differential gene expression observed across our datasets. Biological processes involving the migration, recruitment, and activation of lymphocytes, but also lipid metabolism, were most significantly impacted in our disease model. Many of the identified dysregulated genes and affected biological functions were similarly regulated in the differential expression datasets of other transcriptomic-based studies of SjD patients and mouse models.

**Conclusion:**

Chronic inflammation induces significant alterations to the LG transcriptome, promoting the recruitment of immune cells into the LG and the overactivation of inflammatory responses that severely impede normal secretory function. Future studies targeting some of the discovered key dysregulated molecules/pathways could open up potential therapeutic avenues for the treatment of chronic LG inflammation and secretory hypofunction.

## 1 Introduction

Aqueous-deficient dry eye disease (DED) is primarily caused by insufficient tear and low-grade lacrimal fluid secretion from the lacrimal gland (LG) onto the ocular surface (1). Secretions from the LG provide key proteins, electrolytes, growth factors, and antimicrobial agents needed to protect, nourish, and maintain the normal functions of the cornea and conjunctiva (2–4). LG dysfunction can be induced by several factors, such as inflammation, aging, and other biological and/or environmental factors, resulting in insufficient lacrimal fluid and tear production (1, 2). DED patients often experience severe eye irritation, blurry vision, and even vision-threatening conditions such as corneal ulcers and loss of corneal transparency in severe cases (4).

The main inflammatory factor inducing LG dysfunction and DED is driven by the onset of Sjogren’s disease (SjD) (5), a chronic autoimmune disorder of the lacrimal and salivary glands (SG) that affects an estimated 1–4 million Americans, mostly women, resulting in dry eye and dry mouth (6, 7). Primary SjD, occurring in patients with no other autoimmune diseases, is characterized by focal lymphocytic infiltration of the LG and SG, production of autoantibodies, and increased expression of multiple pro-inflammatory cytokines (1), many of which, as we and others have previously reported, impair LG function and inhibit neurally stimulated LG secretion (8, 9).

Despite extensive research efforts to understand the general pathophysiology and overall factors involved in DED, the underlying mechanisms that lead to insufficient LG secretion due to chronic inflammation of the LG are still not well understood, and thus, current existing treatment modalities just provide temporary relief without addressing such underlying pathophysiological mechanisms (1, 10). Over the last decade, transcriptomic profiling and analysis has emerged as one of the most useful tools in the identification of disease biomarkers and discovery of molecular targets for therapeutic development, with RNA sequencing (RNA-seq) surfacing as a widely used method to analyze the transcriptome by quantifying gene expression at the RNA level through deep-sequencing techniques (11).

Non-obese diabetic (NOD) mice have been extensively used as a model for human primary SjD due to similarities in the pathology of lymphocytic infiltration of the LG that occurs in SjD patients (12–14). In these mice, the degree and development of autoimmune dacryoadenitis is sex-dependent, with males developing it earlier and significantly more severely than females (13–15). Such LG lymphocytic infiltration is absent before 4 weeks of age, with the early observed onset occurring between 6 and 8 weeks of age (11) and increasing in a time-dependent manner, with older (>12 weeks) mice showing substantial disease severity (16). However, because a later development of diabetes in the NOD strain can influence the overall transcriptome and proteome expression profiles, a NOD-derived, diabetes-resistant (NOR) strain is often used to account for this confounding variable. Male NOR/LtJ mice display similar Sjogren-like pathophysiology as characterized in the NOD strain, namely, lymphocytic infiltration of LGs, the production of autoantibodies, and functional secretory hypofunction with significantly reduced stimulated tear output (15, 16). Therefore, we used male NOR mice in this study as a model of chronic LG inflammation-induced DED. BALB/c mice are an established control strain as they do not develop signs of LG inflammation nor reduced tear secretion at the stated ages (16).

Recently, using mass spectrometry-based proteomic analysis, we showed that chronic inflammation induces significant changes in the proteome of LGs from the described SjD animal model (17). Expanding on this proteomic study, and in an effort to quantify autoimmune inflammation-induced changes in the LG at the transcription level, we conducted RNA-seq-based analyses of LGs from male NOR/LtJ mice and their age- and sex-matched BALB/c controls before the onset of LG lymphocytic infiltration and DED (3 weeks) and at the early and developed stages of the disease (8 and 16 weeks, respectively). The present study provides a comprehensive overview of the LG transcriptome profiles and the induced gene expression changes caused by chronic inflammation of the LG.

## 2 Materials and methods

### 2.1 Animals

All procedures were approved by the Tufts Medical Center Institutional Animal Care and Use Committee and were performed in accordance with the Association for Research in Vision and Ophthalmology (ARVO) statement for the use of animals in ophthalmic and vision research. For the diseased animal model, male NOR/LtJ mice (strain #002050) purchased from The Jackson Laboratory (Bar Harbor, ME) and of the following ages—3 (*n* = 3), 8 (*n* = 9), and 16 weeks (*n* = 9)—were used. The respective sex- and age-matched 3-, 8-, and 16-week-old BALB/cJ mice (strain #000651) were used as the wild-type control (*n* = 3, 9, and 9, respectively) and were also purchased from The Jackson Laboratory. Animals were euthanized by gradual carbon dioxide (CO_2_) inhalation followed by cervical dislocation, and their exorbital LGs were excised and processed for RNA extraction or fixed for downstream histology and immunostaining procedures, as described below.

### 2.2 RNA extraction and purification

Excised exorbital LGs were placed in RNAlater Stabilization Solution (Thermo Fisher Scientific, Waltham, MA) for storage at −20°C prior to RNA extraction. Total RNA was extracted and purified using the RNeasy Midi Kit (Qiagen, Germantown, MD) following the manufacturer’s protocol. In brief, a single LG was placed in a 15-mL centrifuge tube and homogenized using a conventional rotor-stator for 45 s in 1 mL of buffer RLT, and then an additional 1 mL of buffer RLT was added and mixed well. The homogenate was centrifuged at 4,000×*g* for 10 min, and the supernatant was collected and transferred to a 15-mL centrifuge tube. Two milliliters (1 volume) of 70% ethanol was added to the homogenized lysate and immediately shaken vigorously. Without delay, the sample was applied to a RNeasy Midi column placed in a 15-mL centrifuge tube and centrifuged for 5 min at 3,000×*g*. Next, 4 mL of buffer RW1 was added to the RNeasy column and centrifuged for 5 min at 3,000×*g*. Then, 2.5 mL of buffer RPE was added to the RNeasy column and centrifuged for 5 min at 3,000×*g*, and this step was repeated once more. Lastly, for elution of the purified RNA, 150 μL of RNase-free water was added to the RNeasy column and allowed to stand for 1 min before centrifuging for 3 min at 3,000×*g*. This elution step was repeated once more by adding 150 μL of RNase-free water to the RNeasy column, and the eluate was combined for higher recovery of purified RNA. Purified RNA samples were stored at −80°C until downstream use.

Total RNA concentration and quality, indicated by the RNA integrity number (RIN), were measured using the 2100 Bioanalyzer system (Agilent Technologies, Santa Clara, CA) and Agilent RNA 6000 Nano Kit, following the manufacturer’s instructions. For the samples analyzed, RIN was generated for each Bioanalyzer trace using the 2100 Expert Software (Agilent Technologies). The maximum RIN score was 10.0, and samples with RIN values of 8.0 or higher were deemed suitable for RNA sequencing analyses.

### 2.3 Library preparation and DESeq2 differential gene expression analysis

High-quality total RNA (100–200 ng) samples (*n* = 3 biological replicates for each NOR and BALB/c age group; 8- and 16-week samples were pooled from two to three age- and strain-matched mice) were used as input for the generation of the RNA-seq libraries using the Illumina TruSeq stranded mRNA kit, following the manufacturer’s instructions. The resulting libraries were analyzed on an Agilent Fragment Analyzer for molar quantification and size distribution. The libraries were then pooled at equal molar and subsequently sequenced on an Illumina HiSeq 2500 sequencer using V4 chemistry and single read 50 bases format. Basecalling and demultiplexing were performed on the raw data using Illumina’s bcl2fastq software, and a compressed (.gz) fastq file was generated for each sample.

Quality control of the read data was conducted using the FastQC tool. Sequencing reads from the compressed fastq files were then mapped to the mouse reference genome (mm10) using the HISAT2 alignment program. The resulting BAM files from the alignment and the mm10 gene annotation files (in GTF format) were then used to create a raw gene count table using the featureCounts program from the Rsubread package (18). Downstream differential expression analyses were conducted using the DESeq2 R/Bioconductor package (19), and the results from each NOR versus BALB/c pairwise comparison were exported as .csv files.

### 2.4 H&E staining

LG samples from at least three different NOR and BALB/c mice were fixed overnight in 4% methanol-free formaldehyde in phosphate-buffered saline (PBS). Glands were then washed three times in PBS and dehydrated using a series of xylene and graded ethanol solutions prior to embedding as per standard procedure. Then, the tissue samples were embedded in paraffin blocks, and tissue sections (7 µm in thickness) were mounted onto Superfrost Plus microscope slides (Thermo Fisher Scientific, Waltham, MA). Sections were then dried in an Isotemp oven at 55°C, deparaffinized in three xylene washes, and rehydrated using a series of graded ethanol and water (running tap and distilled) washes. The slides were then submerged in Harris hematoxylin solution (modified) for 2 min, rinsed briefly in distilled water, and dipped in an acid−alcohol solution prior to being placed in an eosin Y solution (1% alcoholic) for 1 min. Next, the slides were dehydrated and cleared in a series of graded ethanol solutions and three final xylene washes prior to coverslipping with an express, non-aqueous mounting medium.

### 2.5 Immunofluorescence staining

LG tissue sections (6 µm in thickness) from three different NOR and BALB/c mice were prepared, deparaffinized, and rehydrated as described above. Next, antigen unmasking was performed by placing the slides in an antigen retrieval solution (BD Retrievagen B, pH 9.5; BD Biosciences, San Diego, CA, USA) and microwaving them for 10 min. Tissue sections were then allowed to cool down at room temperature (RT) for 30 min and washed in PBS three times for 5 min each. Then, tissue permeabilization was performed by incubation with 0.1% Triton X-100 in PBS at RT for 10 min. After three washes in PBS, non-specific binding sites were then blocked using 10% normal donkey serum (Jackson ImmunoResearch Laboratories, West Grove, PA) for 1 h at RT. Tissue sections were then incubated with anti-STAT1 (1:200, cat. # 82016-1-RR; Proteintech, Rosemont, IL, USA) or anti-TLR9 (1:200, cat. # 27769-1-AP; Proteintech, Rosemont, IL, USA) primary antibodies overnight at 4°C. Primary antibodies were omitted for sections used as negative controls. The following day, tissue sections were incubated with a donkey anti-rabbit IgG H&L, Alexa Fluor 488-conjugated secondary antibody (1:1,000 dilution, Abcam, Waltham, MA, USA) for 1 h at RT. Any endogenous tissue fluorescence was then quenched using Invitrogen’s Ready Probes Tissue Autofluorescence Quenching kit (Thermo Fisher Scientific, Waltham, MA, USA). Prior to coverslipping, a SlowFade Diamond Antifade mounting medium (Thermo Fisher Scientific, Waltham, MA, USA), containing DAPI for nuclear counterstaining, was applied to each section. The immunostained slides were then imaged using a color digital camera (SPOT Insight CMOS; SPOT Imaging, Sterling Heights, MI, USA) mounted on an Eclipse E600 microscope (Nikon Instruments Inc., Melville, NY, USA) and using the same camera parameters for both groups.

### 2.6 Ingenuity pathway analysis of differentially expressed genes

QIAGEN’s Ingenuity Pathway Analysis (IPA) software was used to analyze and interpret the differential gene expression results obtained from each pairwise differential expression analysis (DEA). To focus on the most significant transcriptional changes and investigate the most meaningful altered biological processes in our disease model, in addition to an adjusted *P*-value cutoff (adj. *P*-value ≤ 0.05), an absolute log_2_ fold-change cutoff (|log_2_ fold-change| ≥ 1) was applied on the uploaded DEA datasets. Thus, mapped transcripts with a minimum two-fold change (up or down) and a false discovery rate (FDR)-adjusted *P*-value at or below 0.05 were considered to be differentially expressed and analysis-ready molecules. The SynGO ID conversion tool (20) was used to obtain the Entrez Gene IDs of the listed genes in each data table and increase the number of genes mapped to the QIAGEN Knowledge Base by designating both the “gene symbols” and “Entrez IDs” columns as the input for gene identification during the data upload process. The algorithms developed for the different analysis tools provided in IPA have been described in more detail by Krämer A. and colleagues (21). Once the DEA datasets from the 3-, 8-, and 16-week NOR versus BALB/c pairwise comparisons were successfully uploaded onto the IPA application, an expression-based (expression log ratio) core analysis was performed for each comparison. Next, a comparison analysis was conducted in IPA, which compares the results from the selected individual core analyses and presents them in a heatmap format, to compare and contrast the molecular mediators and biological processes being activated or inhibited at each timepoint and disease stage.

## 3 Results

### 3.1 Development of autoimmune dacryoadenitis leads to major alterations in the LG transcriptome

**Figure 1:**
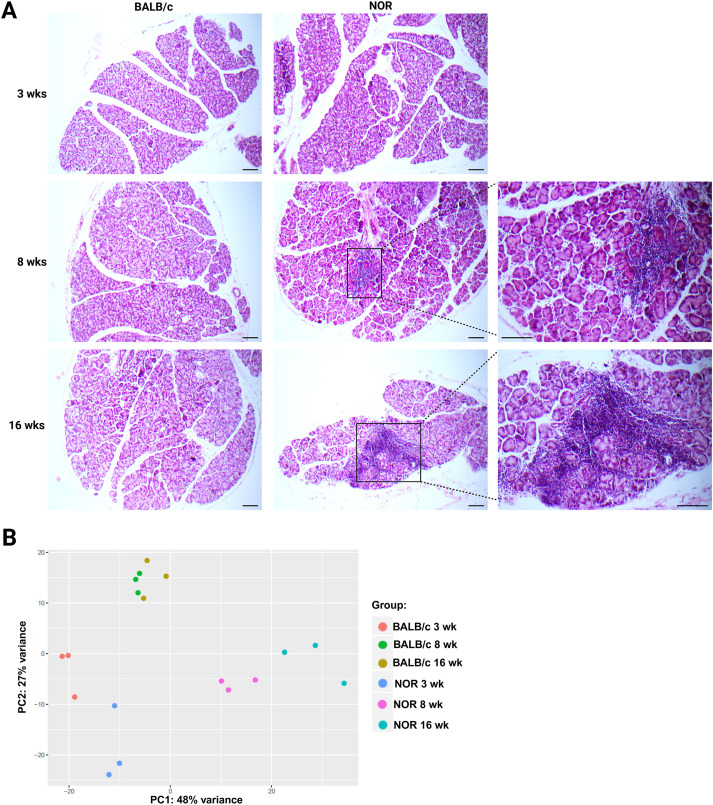
Lymphocytic infiltration of the lacrimal gland (LG) and principal component analysis (PCA). **(A)** Representative H&E staining photomicrographs of exorbital LGs from 3-, 8-, and 16-week-old NOR and BALB/c mice at each age point. *n* = 3–4 stained serial LG sections from at least three separate animals per strain/age group. Scale bars = 100 µm. Lymphocytic infiltration of the LG was absent at 3 weeks in both NOR and BALB/c mice. Mild infiltration was observed at 8 weeks in the glands of NOR animals. Meanwhile, extensive lymphocytic foci were present in the LGs of NOR mice at 16 weeks, which was still absent across all age-matched BALB/c controls. **(B)** PCA plot of LG samples (*n* = 3 biological replicates) used for RNA-seq and subjected to DESeq2 differential gene expression analysis. Biological replicates within each strain age group were relatively clustered together. There was a clear separation between the LG samples from 8- (early disease stage) and 16-week (developed disease stage) NOR mice and the pre-disease (3-week) NOR samples, as well as all three BALB/c control groups.

Focal lymphocytic infiltration of the LG is one of the major hallmarks underpinning the pathogenesis of Sjogren’s DED. The gland-infiltrating lymphocytes consist primarily of CD4^+^ helper T cells, CD8^+^ cytotoxic T cells, and B cells; however, the presence of other leukocytes such as plasma cells, macrophages, mast cells, and dendritic cells has also been reported in affected glands (22, 23). Aligning with previous studies, the early disease stage and its pathophysiological manifestation in NOD/NOR mice were considered to be at 8–12 weeks of age, while mice of ages between 12 and 20 weeks were considered to be at a moderately advanced disease stage (15, 16). Consistent with these considerations, hematoxylin–eosin (H&E) histological staining of LGs from diseased and control animals showed the absence of such glandular lymphocytic infiltration at 3 weeks of age, with some LG areas from NOR animals beginning to be infiltrated by 8 weeks (**Figure 1A**). Significant lymphocytic infiltration was observed in the LGs of 16-week-old NOR mice when compared to age- and sex-matched BALB/c controls and to young (3-week-old) NOR mice (**Figure 1A**). Therefore, LGs from 3-, 8-, and 16-week diseased NOR and control BALB/c mice were used to represent the pre-onset, early, and moderately severe stages of autoimmune dacryoadenitis and DED in these mice, respectively.

As expected, principal component analysis (PCA) of the gene expression profiles of all LG samples (**Figure 1B**) revealed a clear separation between the NOR and BALB/c samples, highlighting how inflammation of the LG leads to strong alterations at the transcriptional level. Notably, transcriptomic data from the 8- and 16-week BALB/c samples clustered together and away from the 3-week BALB/c, suggesting that no major expression differences are taking place in the LGs of control animals between the 8- and 16-week timepoints. Transcriptomic profile differences between the very young (3-week) and older (8- and 16-week) BALB/c LG samples may be due to gland development differences and when the LG has reached maturity in these animals. Conversely, there was a separation between the transcriptomic data of 8- and 16-week NOR samples, indicating a continuous shift in gene expression as the degree of LG inflammation increased. The separation from the pre-disease, 3-week NOR samples increased within the strain as they aged, corroborating the described development and progression of chronic LG inflammation in this murine model.

### 3.2 Differentially expressed genes between NOR versus BALB/c LGs as inflammation progresses

**Figure 2:**
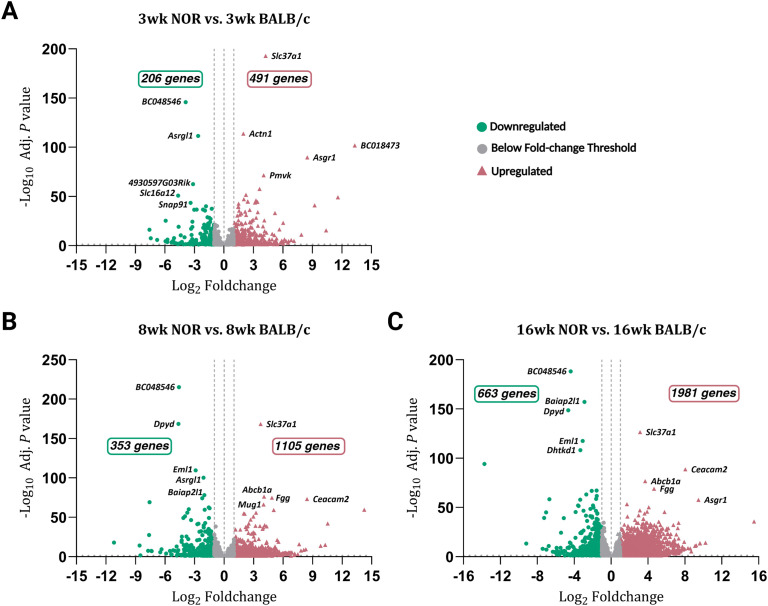
Volcano plots of differentially expressed genes (DEGs) across all three pairwise comparisons. Volcano plots (log_2_ fold-change on the *x*-axis, −log_10_ adjusted *P*-value on the *y*-axis) with defined fold-change cutoffs (absolute log_2_ fold-change ≥ 1) displaying DEGs (those with at least a 2-fold increase or decrease and an adjusted *P*-value ≤ 0.05) across the three conducted pairwise comparisons. **(A)** At 3 weeks, 697 genes (206 downregulated and 491 upregulated) were identified to be differentially expressed between the LGs of NOR mice compared to control samples. A total of 1,458 genes (353 downregulated and 1,105 upregulated) **(B)** and 2,644 genes (663 downregulated and 1,981 upregulated) **(C)** were differentially expressed at 8 and 16 weeks, respectively. The top 5 most significantly up- and downregulated genes (based on the adjusted *P-*values) are selectively labeled on the plots. Dots (in green) depict transcripts that are downregulated, while triangles (in red) depict those upregulated. Gray dots represent those genes that did not pass the set fold-change cutoffs described above. Volcano plots were generated using GraphPad Prism 10.

Differential gene expression was evaluated between the diseased and control LGs across three separate pairwise comparisons. Differentially expressed genes (DEGs) between 3-, 8-, and 16-week NOR versus age-matched BALB/c LGs were graphically visualized using volcano plots with defined adjusted *P*-value and fold-change cutoffs (adjusted *P*-value threshold = 0.05; absolute log_2_ fold-change threshold = 1). A total of 697 genes (206 downregulated and 491 upregulated) passed the set cutoffs and were identified as differentially expressed between NOR and BALB/c LGs during the pre-disease stage (3 weeks) (**Figure 2A**). At 8 weeks, 1,458 genes were identified as differentially expressed (353 downregulated and 1,105 upregulated) (**Figure 2B**). Meanwhile, 2,644 genes (663 downregulated and 1,981 upregulated) were found to be differentially expressed between the disease and healthy samples at 16 weeks (**Figure 2C**). Overall, the number of DEGs between NOR LGs and BALB/c controls increased in accordance with the progression and severity of leukocyte infiltration of the LG, indicating that the heightened presence of immune cells in the more acute diseased LGs induces or inhibits the expression of a substantial number of genes when compared to less severely or non-inflamed glands. The complete, unprocessed differential expression analysis dataset for each of the three pairwise comparisons is provided as supplemental material (**Supplementary Tables 1**-**3**).

### 3.3 Significantly enriched canonical pathways during LG inflammation

**Figure 3:**
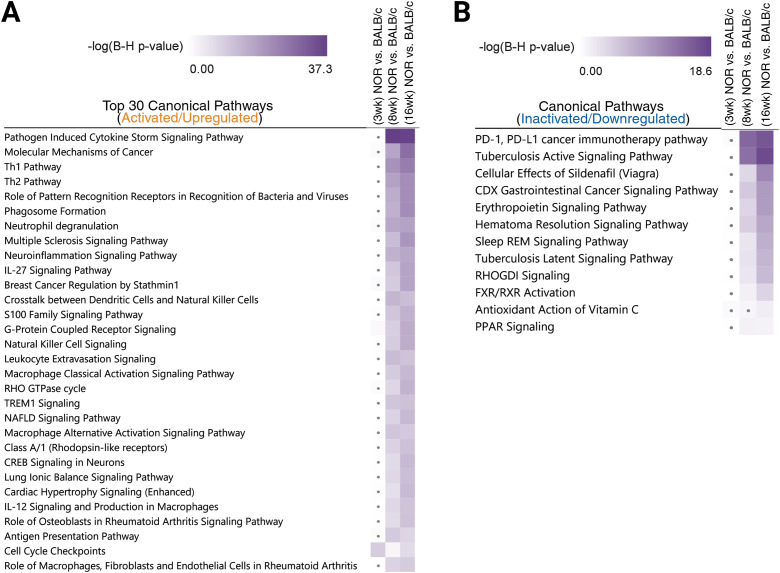
Comparison of enriched canonical pathways between all three timepoints and disease stages. The pathways tool in the Ingenuity Pathway Analysis (IPA) software (QIAGEN) was used to identify the most significantly overrepresented canonical pathways across the datasets from all three pairwise comparisons. **(A)** Comparison heatmap of the top 30 most significantly (based on the −log_10_ Benjamini–Hochberg, BH-adjusted *P-*values) activated/upregulated (*z*-score ≥ 2) canonical pathways. **(B)** All 12 canonical pathways significantly (BH-adjusted *P-*value ≤ 0.05 or ≥1.3, if in the −log form) predicted to be inactivated/downregulated (*z*-score ≤ 2) across our differential expression datasets. Pathways not significantly enriched (below the adjusted *P*-value threshold) at a particular timepoint appear white and/or are marked with a “dot” in the heatmap square.

To examine the potential cellular and/or metabolic pathways altered during chronic inflammation of the LG that occurs in SjD, we analyzed each DEG dataset using the canonical pathways tool in IPA. Particularly, the most significantly enriched pathways during the early (8 weeks) and more advanced (16 weeks) stages of LG inflammation in NOR mice were under broader pathway categories such as “cellular immune response,” “cytokine signaling,” “humoral immune response,” and “immune system,” which were not observed across the dataset of DEGs identified at the pre-disease (3-weeks) stage (**Supplementary Figures 1**–**3**). The overrepresentation of these specific pathway categories points to a dysregulated immune response, especially that mediated by the adaptive immune system. To better visualize and compare the pathways enriched across our three pairwise comparisons, we used the IPA comparison analysis feature. The top 30 most significantly (based on their adjusted *P-*values) activated (with *z*-scores ≥ 2) canonical pathways are selectively presented in **Figure 3A**. Notably, DEGs between LGs of diseased NOR mice and BALB/c controls were most significantly associated with the activation of cytokine-mediated signaling pathways such as the “pathogen-induced cytokine storm signaling” and the pathways leading to the differentiation of naive CD4^+^ T cells into activated T helper (Th) cells like Th1 and Th2. The upregulation of genes in our datasets like *Ifng* (interferon gamma), *Tnf* (tumor necrosis factor alpha), and interleukins (IL) such as *Il12* and *Il33*, all of which are key mediators of the cytokine storm signaling and/or Th1/Th2 activation pathways, highlights the critical role of cytokine signaling in mediating the inflammatory responses and autoimmune conditions observed in patients and animal models of SjD.

The list of pathways positively enriched or upregulated was significantly greater than that of the pathways predicted to be inhibited or downregulated in the inflamed LGs in comparison to healthy controls. Among the 12 pathways significantly predicted to be inhibited in our comparison analysis (**Figure 3B**) were the FXR/RXR (Farnesoid X and retinoid X receptor) activation pathway and the PPAR (peroxisome proliferator-activated receptor) signaling pathway. In particular, FXR has been reported to regulate immune cell activities and modulate anti-inflammatory responses (24, 25). Similarly, PPARs have been demonstrated to exert important anti-inflammatory activities (26–28), with PPARγ expression, in particular, found to be constitutively reduced in the salivary epithelia of SjD patients (28).

### 3.4 Significant upregulation of cytokines and transcription factors as the major drivers of gene expression changes during LG inflammation

**Figure 4:**
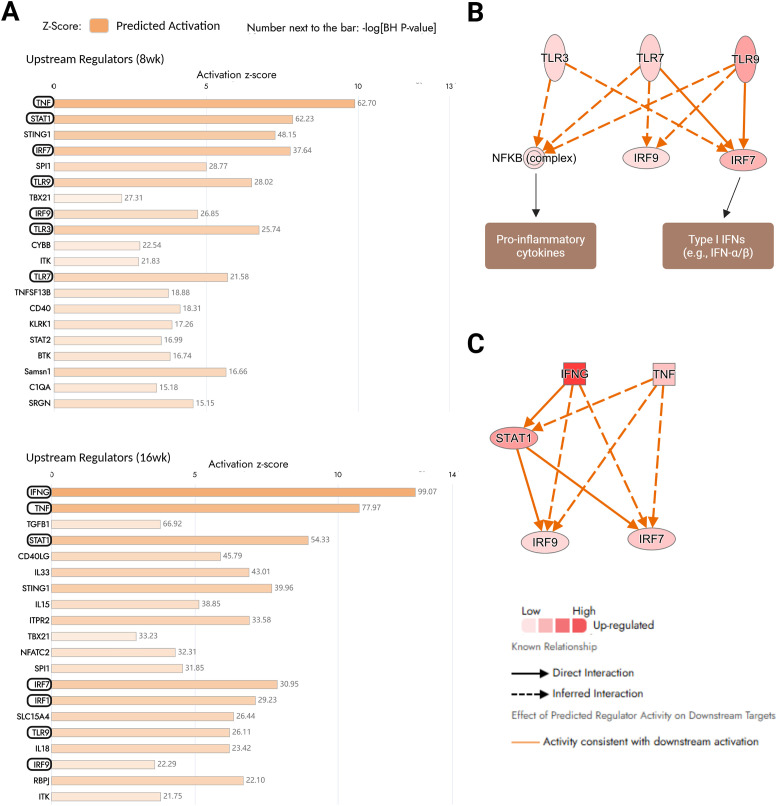
Differentially expressed genes (DEGs) in the dataset acting as potential “upstream” regulators. DEGs in our dataset acting as “upstream” regulators and potentially driving the significant gene expression changes observed in the diseased lacrimal glands (LGs) were evaluated using the “upstream regulator” analysis tool in the IPA software. **(A)** Top 20 dataset molecules predicted to majorly induce the gene expression changes in the diseased LGs during the early (8 weeks, top bar graph) and more advanced (16 weeks, bottom bar graph) stages of LG inflammation and hypofunction. Highly upregulated cytokines in the differential gene expression datasets such as *Tnf* (tumor necrosis factor alpha) and *Ifng* (interferon gamma); several transcription factors such as *Stat1* (signal transducer and activator of transcription 1), *Irf7*, or *Irf9* (interferon regulatory factors, IRFs); and toll-like receptors (TLRs) like *Tlr9* were most significantly predicted to drive the transcriptional changes observed in this study. The −log BH-adjusted *P*-values for the significance of each predicted “upstream” regulator are placed next to each bar. **(B)** Molecular mechanistic network of how upregulated/activated “upstream” regulators like TLRs and IRFs can work together as a network to elicit further gene expression changes, especially the upregulation of pro-inflammatory cytokines and interferons. **(C)** Molecular mechanistic network of how the activated/upregulated expression of these cytokines can also elicit further downstream transcriptional changes by activating other transcriptional factors.

Potentially activated or inhibited upstream molecules/regulators responsible for driving the transcriptional changes observed in the diseased LGs when compared to normal controls were investigated using the Upstream Regulators analysis tool in IPA. To better examine the most significant molecular regulators potentially driving the disease in our murine model, we focused on those upstream regulators which predicted activation or inhibition state, matched the differential expression (up- or downregulated and by at least two-fold) observed in our pairwise comparisons. The top 20 differentially expressed molecules predicted to majorly induce the gene expression changes in our model during the early (8 weeks) and more developed (16 weeks) stages of LG inflammation are presented in **Figure 4A**. The upregulated expression of pro-inflammatory cytokines such as TNF and IFNG was predicted to most significantly drive the gene expression changes observed in the diseased LGs, corroborating the role of cytokine-mediated signaling pathways in the development of chronic LG inflammation and highlighting these two cytokines as key biomarkers for disease progression. Additionally, among the top-most molecules predicted to induce these transcriptional changes, several transcription factors including *Stat1* (the most significant under this molecule type), *Irf7*, *Irf1*, *Irf9*, *Spi1*, and *Tbx21*, and toll-like receptors (*Tlr9*, *Tlr3*, and *Tlr7*) were also identified. TLR9, TLR3, and TLR7 are generally considered endosomal TLRs, and their activation can lead to the increased production of various pro-inflammatory cytokines through the downstream activation of nuclear factor kappa B (NF-κB) and/or regulate the transcription of type I interferons through the activation of interferon-regulatory factors (IRFs) such as IRF7 (**Figure 4B**) (29, 30). Endosomal TLR trafficking and processing and several other TLR-related pathways were indeed significantly enriched in our canonical pathway analysis (**Supplementary Figure 4**), suggesting that the signaling cascades mediated by these transmembrane receptors may play an important role in the overactive inflammatory responses occurring in the LGs of NOR mice. Furthermore, the significant overexpression of genes encoding transcription regulators such as STAT1 and IRF9, which are part of signal transduction pathways like the JAK/STAT signaling pathway, points to the active transmission of signals from cell surfaces to the nucleus, which when induced by inflammatory stimuli such as IFNG and TNF (**Figure 4C**) can lead to notable transcriptional changes, further modulating the chronic immune and inflammatory responses observed in the diseased LGs.

**Figure 5:**
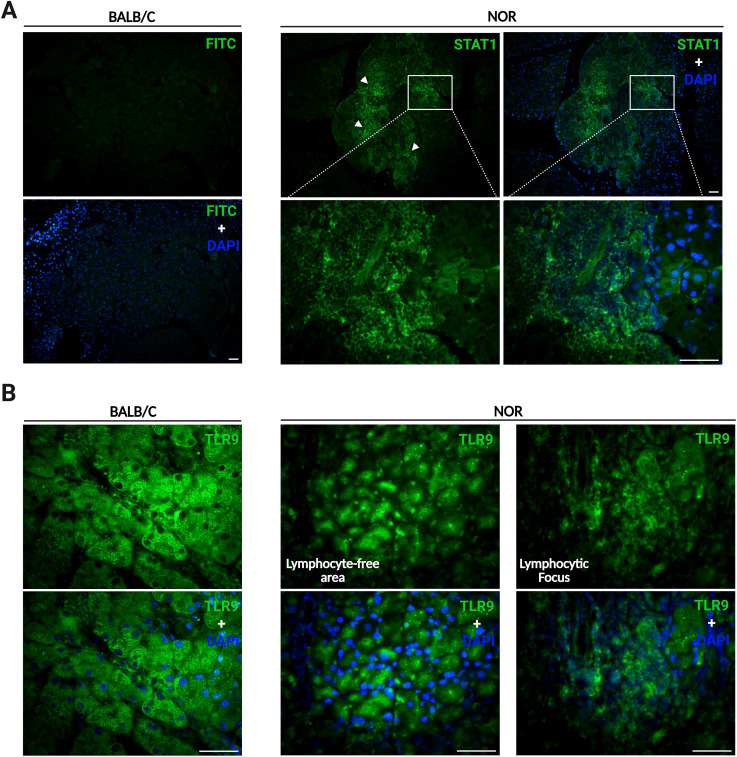
Differential spatial expression analysis of selected differentially expressed genes (DEGs) using immunofluorescence staining. The differential gene expression of selected molecules identified to act as major “upstream” molecular regulators driving the transcriptomic changes in the diseased lacrimal glands (LGs) was evaluated in immunostained LGs from 16- to 21-week-old NOR and BALB/c mice (*n* = 3 for both groups). **(A)** Representative immunofluorescence photomicrographs of signal transducer and activator of transcription 1 (STAT1) staining in BALB/c (left-side panels) and NOR (right-side panels) LG tissue sections. Arrowheads depict strong positive STAT1 staining in areas where lymphocytic foci are present in LG tissue sections of NOR mice. A higher magnification view of a lymphocytic focus is shown on the bottom right panels. Scale bars = 50 µm. **(B)** Representative immunofluorescence photomicrographs of toll-like receptor 9 (TLR9) staining in BALB/c (left-side panels) and NOR (right-side panels) LG tissue sections. TLR9 was also strongly detected in the lymphocytic infiltrates present in the LGs of NOR mice (right-side panels). Scale bars = 50 µm.

The upregulation of selected upstream molecules, such as STAT1 and TLR9, identified to significantly drive the transcriptional changes occurring in the chronically inflamed LGs, was confirmed by immunofluorescence staining. Notably, STAT1 expression was highly detected specifically in the lymphocytic foci present in the LGs of NOR mice, which is absent in the control BALB/c LGs (**Figure 5A**). The pattern recognition receptor, TLR9, was highly expressed in the epithelium of both the diseased and control LGs; however, similar to the expression of STAT1, the expression of TLR9 was also highly detected in regions with substantial lymphocytic infiltration in the LGs of NOR mice (**Figure 5B**). The high expression of both STAT1 and TLR9 in the lymphocytic infiltrates heavily present in diseased LGs from NOR mice, but absent in healthy glands from BALB/c animals, most likely explains the significant upregulation of STAT1 and TLR9 identified in our differential gene expression analysis.

### 3.5 LG inflammation-induced transcriptional changes mainly impact biological processes involved in immune responses, but also in lipid metabolism and ion mobilization

**Figure 6:**
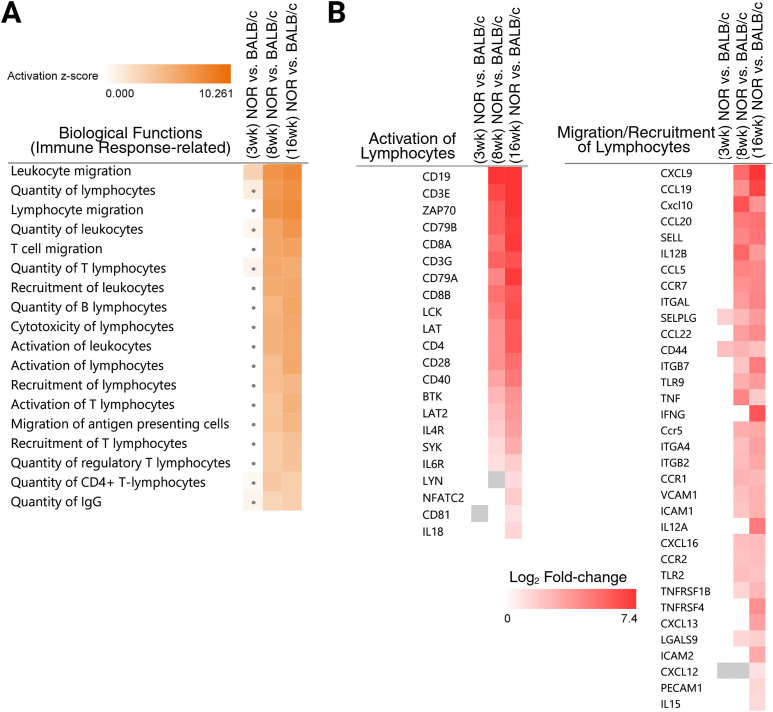
Comparison heatmap of altered biological functions related to a heightened lymphocytic activity. Biological processes/functions significantly impacted by the differential gene expression patterns identified in the diseased LGs when compared to control samples were assessed and compared across the three studied timepoints. **(A)** Heatmap comparing the activation of some selected biological functions, which were mainly related to an increased lymphocytic activity and immune response, across the 3-, 8-, and 16-week NOR versus BALB/c comparisons. Individual biological functions not significantly activated (with *z*-scores not above the ≥ 2 threshold) at a particular timepoint are shown with a “dot” mark in the heatmap square. **(B)** Heatmap comparing the differential expression of selected genes (red squares depict upregulation) that play key roles in the activation (left side heatmap) and the overall migration/recruitment (right side heatmap) of lymphocytes. Genes that were not differentially expressed between the LGs of NOR mice compared to BALB/c controls at a specific timepoint have an empty or “white” heatmap square space. A gray heatmap square depicts that the gene was in the differential expression dataset but did not pass the set fold-change cutoff (absolute log_2_ fold-change ≥ 1). Heatmaps were generated using the IPA “diseases and functions” comparison analysis tool.

The effects of the gene expression changes observed in the diseased LGs across our pairwise comparisons were further evaluated using the “diseases and functions analysis” tool in IPA. As expected, the most significantly affected downstream biological processes or functions were associated with the immune system and inflammatory responses. Specifically, functions involving the recruitment, migration, and activation of lymphocytes were repeatedly predicted to have significantly increased (both at 8 and 16 weeks), given the observed transcriptional changes in our experimental datasets (**Figure 6A**). The temporal differential expression of dataset genes encoding some of the crucial molecules involved in the activation of lymphocytes, including T-cell receptor-cluster of differentiation 3 (CD3) complex molecules such as *Cd3e* (epsilon subunit) and *Cd3g* (gamma subunit); T-cell co-receptors such as *Cd8a* and *Cd8b* (CD8 alpha and beta chains), *Cd4*, and *Cd28*; B-cell co-stimulatory molecules like *Cd19*, *Cd40* (and its ligand *Cd40lg*), and *Cd81*, as well as other important molecules needed for the activation and development of T or B lymphocytes such as *Lck*, *Zap70*, *Lat*, *Lat2*, *Nfatc2*, *Lyn*, *Cd79a/Cd79b*, *Syk*, *Btk*, *Il4r*, and *Il6r*, is presented in **Figure 6B** (left-side heatmap).

Similarly, a heatmap displaying the temporal upregulated expression level of genes encoding some of the key molecules contributing to the migration and recruitment of lymphocytes is also presented in **Figure 6B** (right-side heatmap). This includes genes encoding selectin-related molecules such as *Sell* and *Selplg*; integrins such as *Itgb7*, *Itgb2*, *Itga4*, *Itgal*, *Itgb1*, and other adhesion molecules like *Icam1*, *Icam2*, *Vcam1*, and *Pecam1*; chemokines and their receptors such as *Ccl19*, *Ccl5*, *Ccl20*, *Cxcl9*, *Cxcl10*, *Ccr7*, *Ccr5*, and *Ccr1* (among others); cytokines such as *Il12b*, *Il12a*, *Il15*, *Tnf*, and *Ifng*; and other molecules like *Tlr9*, *Tlr2*, *Tlr1*, *Tnfrsf1b*, *Tnfrsf4*, and *Cd44*. As shown in the heatmaps, most of the genes presented were not differentially expressed at 3 weeks, and their expression levels often increased from the 8- to the 16-week timepoint, which is in line with the progression of inflammation observed histologically in this murine model.

**Figure 7:**
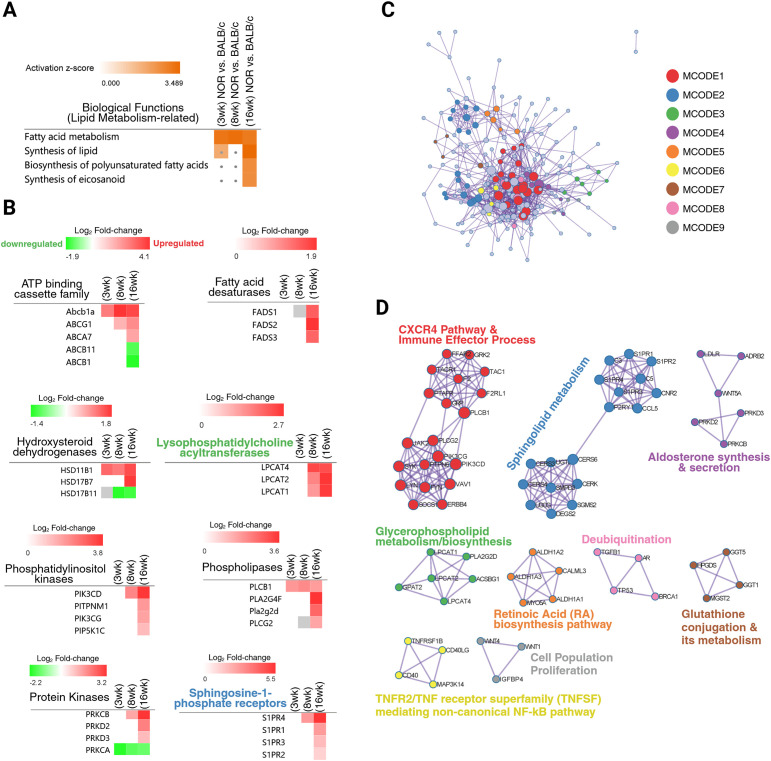
Additional impacted biological functions suggest altered lipid biosynthesis/metabolism in the diseased lacrimal glands. **(A)** Heatmap comparing the significant (B-H adjusted *P*-value ≤ 0.05) activation (*z*-score ≥ 2) of impacted biological processes related to lipid synthesis and metabolism. Particular biological functions not affected or not significantly predicted to be activated at a specific timepoint comparison are marked with a dot in the heatmap. **(B)** Heatmaps comparing the differential expression of multiple genes encoding molecules that belong to the same family/group and that were predicted to positively impact lipid synthesis processes. Red and green heatmap colors represent upregulation and downregulation, respectively, of that particular gene across the compared differential expression datasets. Genes not differentially expressed at a particular timepoint have an empty or “white” heatmap square space. A gray heatmap square depicts that the gene was in the differential expression dataset but did not pass the set fold-change cutoff (absolute log_2_ fold-change ≥ 1). Heatmaps were generated using the IPA “diseases and functions” comparison analysis tool. **(C)** Protein–protein interaction network map of all 234 differentially expressed genes involved in the “synthesis of lipid” biofunction (based on the IPA curated Knowledgebase). **(D)** Identified and separated densely connected clusters based on functional similarities. The most significantly enriched ontology or pathway term for each cluster is shown. The protein–protein network map and term enrichment annotation presented in **(C, D)** were generated using Metascape (metascape.org).

Various lipid biosynthesis/metabolism-related processes were also presently predicted to increase in our analysis, particularly the biosynthesis of polyunsaturated fatty acids such as eicosanoids, in LGs with more advanced (at the 16-week timepoint) autoimmune dacryoadenitis (**Figure 7A**). Altered lipid metabolism and homeostasis has been previously identified and reported in the LGs of Sjogren-associated DED murine models (31–34). Several genes encoding molecules that participate in the synthesis of different eicosanoid subtypes were dysregulated in our DEA dataset. Specifically, genes encoding three out of the four prostaglandin E_2_ (PGE_2_) G protein-coupled receptors (35) (*Ptger1*, *Ptger2*, and *Ptger4*) were upregulated at both the 8- and 16-week pairwise comparisons. Additionally, genes such as *Tbxas1* (thromboxane A synthase 1) and *Tbxa2r* (thromboxane A2 receptor), which encode molecules involved in the synthesis of thromboxane A_2_ (another main prostanoid/eicosanoid member), were also significantly upregulated in the 16-week NOR LGs. The differential expression results of these genes and others involved in the synthesis of different eicosanoid/prostaglandin subtypes are selectively presented in **Supplementary Table 4**.

While investigating the DEGs (234 transcripts, provided in **Supplementary Table 5**) reported to significantly increase lipid synthesis activities in the diseased LGs at 16 weeks (as shown in **Figure 7A**), we identified several genes encoding multiple molecules belonging to the same family/subclass. Specifically, the analyzed genes encoding various ATP-binding cassette subfamily members, some of which are known to act as lipid transporters (34), were differentially expressed in our dataset: *Abca7*, *Abcb1a*, and *Abcg1* were upregulated, while *Abcb1* and *Abcb11* were downregulated (**Figure 7B**). Other DEGs encoding multiple molecules that fall under the same group and are predicted to impact lipid synthesis included the following: fatty acid desaturases 1–3 (*Fads1*, *Fads2*, and *Fads3*), hydroxysteroid dehydrogenases (*Hsd11b1*, *Hsd17b11*, *Hsd17b7)*, lysophosphatidylcholine acyltransferases (*Lpcat1*, *Lpcat2*, *Lpcat4*), phosphatidylinositol kinases (*Pik3cd*, *Pik3cg*, *Pip5k1c*, and the gene for the phosphatidylinositol transfer protein membrane associated 1 molecule, *Pitpnm1*), phospholipases (*Pla2g2d*, *Pla2g4f*, *Plcb1*, and *Plcg2*), protein kinases (*Prkca*, *Prkcb*, *Prkd2*, and *Prkd3*), and sphingosine-1-phosphate receptors (*S1pr1*, *S1pr2*, *S1pr3*, and *S1pr4*) (the temporal differential expression of these genes is shown in the heatmaps presented in **Figure 7B**). The gene expression level of *Abcg1* has been shown to be significantly higher (5.20-fold increase) in the LGs of 12-week-old male NOD mice when compared to matched BALB/c controls (34) and also upregulated in injured murine LGs injected with IL-1α in comparison to uninjured glands (32). Additional DEGs in our analysis encoding important molecules that participate in the transport and homeostasis of cholesterol and/or other lipid-mediated signaling processes, and whose upregulated expression in diseased or injured LGs has also been reported in SjD mouse models, such as *Apoe*, *Ldlr*, and *Ptafr* (32, 34), were also present in our datasets (**Supplementary Figure 5**). Taken together, these findings suggest that these lipid transporter/signaling encoding genes are highly modulated in response to inflammatory stimuli.

To further characterize and examine the class of lipids and potential lipid-mediated biological processes being altered by the up- or downregulation of the DEGs involved in the increased “synthesis of lipid” biofunction, the list of 234 genes was uploaded to Metascape (metascape.org), and protein–protein interaction enrichment analysis was conducted through interrogation of several databases (STRING, BioGrid, OmniPath, InWeb_IM) (36). The resulting molecular physical interactions (physical score > 0.132) formed are shown in a network map (**Figure 7C**). Consistent with the results obtained from the IPA diseases and function comparison analysis, the overall topmost enriched pathways or ontology terms in the network were mainly related to the biosynthesis and metabolism of lipids (not shown). Densely connected network components with functional similarities were further identified and separated into independent clusters by the Molecular Complex Detection (MCODE) algorithm. The top 3 enriched terms for the resulting nine separate clusters are shown in **Figure 7D**. Notably, the most densely connected cluster (red-colored nodes) contained dataset genes associated with cellular-mediated immune response processes, reinforcing the idea of a close interplay between lipid metabolism and the development and/or progression of chronic LG inflammation. Also notably, the second most (blue-colored nodes) and third most (green-colored nodes) highly connected clusters were most significantly enriched in functions related to the metabolism of sphingolipids and glycerophospholipids, respectively. This suggests that in addition to the altered lipid efflux and cholesteryl ester deposition identified in the inflamed LGs of Sjogren-related DED mice (34), changes in glycerophospholipid and sphingolipid-based activities may also be occurring in these glands. However, further lipidomic-based studies are needed to functionally validate these lipidome alterations in diseased LGs and elucidate how these lipid metabolic changes may be contributing to autoimmune LG inflammation and hypofunction.

Lastly, in addition to processes directly involved in activating and mounting immune responses or regulating the biosynthesis of lipids and lipid-based cellular signaling processes, several functions involving the flux/mobilization of cations, mainly Ca^2+^, were also predicted to increase in the diseased 8- and 16-week NOR LGs when compared to their age-matched healthy controls (**Supplementary Figure 6**). Many of the DEGs (especially the multiple upregulated chemokines and cytokines) in our dataset can modulate the mobilization of Ca^2+^ (**Supplementary Figure 6**) and, thus, greatly influence intracellular signaling processes, as Ca^2+^ plays critical roles as a second messenger molecule and is crucial for the activation and function of T and B lymphocytes (37, 38). Therefore, the differential expression of this set of genes known to increase the mobilization and overall flux of Ca^2+^ highlights another important mechanism (enhanced calcium signaling), potentially modulating the overactive immune and inflammatory responses occurring in the LGs of our Sjogren-associated DED mouse model.

### 3.6 Confirmation of differential gene expression results using multi-omics comparison analysis

**Figure 8:**
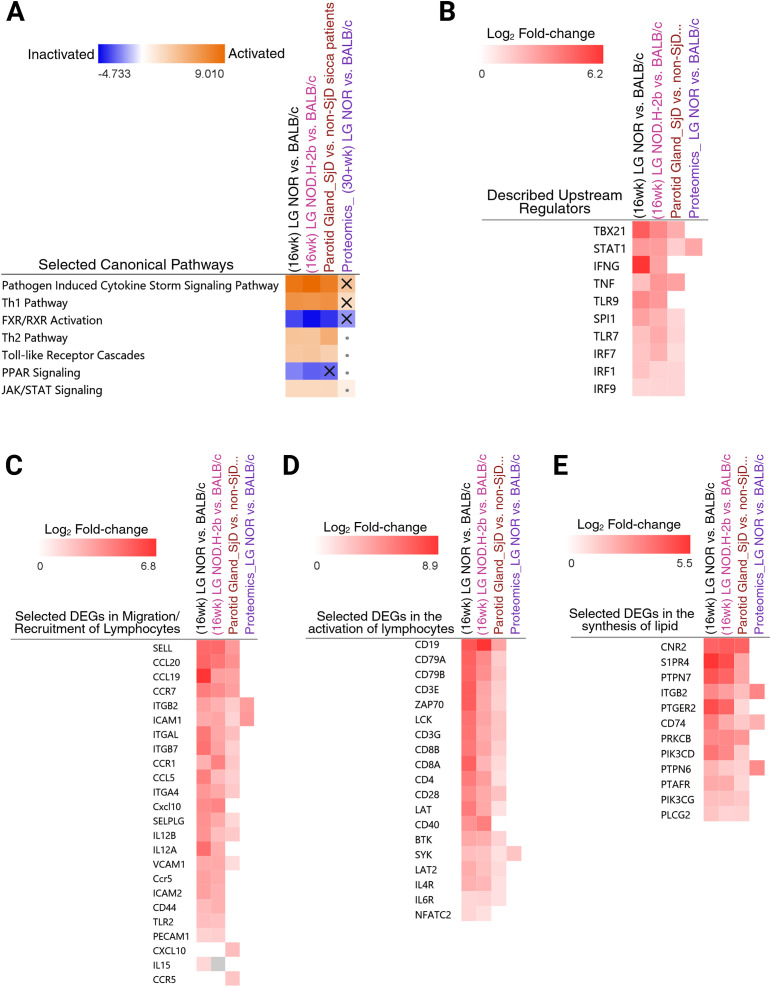
Confirmation of selected enriched canonical pathways, predicted “upstream” regulators, and dysregulated dataset genes. **(A)** Heatmap comparing the predicted activation or inactivation of selected canonical pathways that were significantly enriched in our analysis. All of the presented pathways were also significantly predicted to be activated (*z*-score ≥ 2, orange-colored squares) or inhibited (*z*-score ≤ 2, blue-colored squares) based on the transcriptional changes identified in diseased lacrimal glands (LGs) of another Sjogren’s disease (SjD) mouse model (NOD.H-2b mice) when compared to BALB/c controls. A similar activation or inactivation activity was also predicted when comparing our differential gene expression dataset (for the 16-week pairwise comparison) to differentially expressed genes reported between the parotid salivary glands of SjD-positive versus SjD-negative sicca patients. Pathways not significantly predicted to be activated are marked with a “dot,” while those pathways not significantly enriched in a particular dataset are marked with an “x.” **(B)** Heatmap showing the differential expression pattern of key discovered genes acting as “upstream” regulators driving the general gene expression changes in the diseased LGs. Most of these genes were also similarly upregulated (red-colored squares) in the different transcriptomic datasets used for comparison. Heatmaps comparing and confirming the differential gene expression (upregulation) of many key identified genes contributing to the migration and recruitment of lymphocytes **(C)**, lymphocyte activation **(D)**, and those involved in the synthesis of the lipid network **(E)**.

To confirm the differential expression of the identified genes presented in this RNA-seq-based transcriptomic analysis, we sought to compare our differential gene expression results with previously published, publicly available transcriptomic datasets from studies comparing the transcriptome changes in diseased LGs from NOD.H-2b mice (another mouse model of SjD and dacryoadenitis) versus BALB/c controls (33), the transcriptional changes in human parotid SGs from patients with a positive biopsy for primary SjD versus biopsy-negative non-SjD sicca patients (39), and our published study on the proteomic changes between the LGs of diseased NOR mice versus BALB/c controls (17). As shown in the heatmap presented in **Figure 8A**, the activation of highlighted canonical pathways, such as the “pathogen-induced cytokine storm signaling,” Th1 and Th2 pathways, the signaling cascade mediated by TLR, and the activation of the JAK/STAT signaling pathway, was also confirmed (at the mRNA level) to predictively occur in the LGs of the compared alternative SjD mouse model and in the parotid SGs of SjD-positive patients when compared to their respective healthy counterparts.

Similarly, the upregulation of select DEGs in our dataset predicted to act as the major upstream regulators driving the differential expression, such as *Tnf*, *Stat1*, *Tbx21*, *Tlr7*, and several IRFs, was also observed in the compared transcriptomic analyses—with STAT1, in particular, also found to be upregulated at the protein level in the chronically inflamed LGs (**Figure 8B**). Heatmaps comparing and confirming the upregulation of select transcripts involved in the described increased migration and recruitment of lymphocytes, lymphocyte activation, and lipid synthesis, across the mentioned SjD mouse and human differential expression datasets, are shown in **Figures 8C–E**, respectively. Notably, the upregulated expression of the presented molecules such as ICAM1 (intercellular adhesion molecule 1), ITGB2 (integrin subunit beta 2), and SYK (spleen associated tyrosine kinase) was similarly observed at the protein level in chronically inflamed LGs from NOR mice when compared to healthy controls (last heatmap column in **Figures 8C, D** with text highlighted in purple). The complete list of DEGs (in the 16-week pairwise comparison) for which the downstream translated protein molecules were commonly identified to be dysregulated in our previous proteomic study of diseased NOR LGs versus BALB/c controls (17) is provided in **Supplementary Table 6**. Together, and in addition to confirming the dysregulated expression of the emphasized genes, these comparison analyses also reinforce the usefulness of the presented SjD mouse models in understanding and investigating the underlying mechanisms of Sjogren-associated exocrine gland dysfunction, as many of the detected transcriptional changes closely translate to those observed in human patients.

### 3.7 Potential molecular mechanistic networks of DEGs contributing to increased lymphocyte homing and activity in the diseased LGs

**Figure 9:**
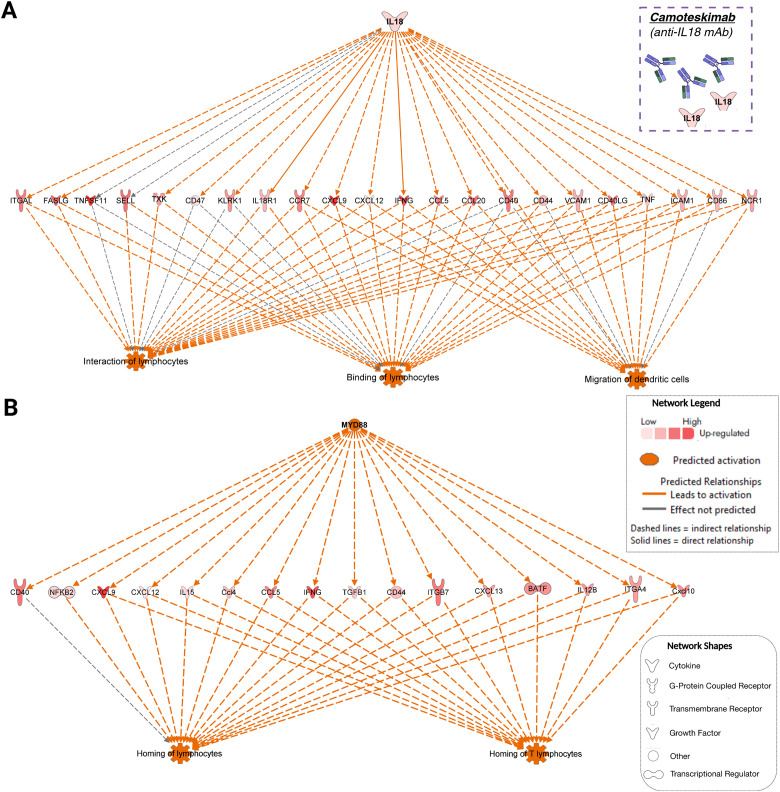
Potential molecular mechanisms contributing to the enhanced infiltration of lymphocytes and autoimmune response observed in the diseased lacrimal glands. **(A)** Molecular mechanistic network depicting how the upregulation of IL-18 (interleukin 18) (shown at the top of the network as an “upstream” regulator) can induce the downstream upregulation or activation of several molecules (upregulated dataset genes, shown at the middle of the network) that have been previously reported to increase lymphocyte cell-to-cell interactions, lymphocyte binding, or the migration of other immune cells such as dendritic cells. **(B)** Mechanistic network depicting how activated MYD88 (myeloid differentiation primary response 88) can induce the upregulation of multiple dataset molecules that have been demonstrated to increase the homing of lymphocytes. Mechanistic networks and molecule-to-molecule predicted activity relationships were algorithmically generated from manually curated scientific literature findings in the IPA Knowledgebase.

We next sought to examine the potential underlying molecular mechanisms impacting biological processes that are key characteristic features of Sjogren-associated LG inflammation by algorithmically connecting “upstream” molecular regulators to DEGs in our dataset that have been reported to affect the downstream biological function or phenotype. An important “upstream” regulator that was repeatedly found in many of the computed mechanistic networks was the interleukin *Il18*. A mechanistic network depicting how the activation or upregulation of *Il18* (log_2_ fold-change = 1.46, adj. *P*-value = 0.01 at 16 weeks) in our disease model affects the expression of DEGs in our dataset, which may increase biological functions such as the interaction and binding of lymphocytes and the migration of dendritic cells, is shown in **Figure 9A**. Additionally, another activated or upregulated (log_2_ fold-change = 0.33, adj. *P*-value = 0.03 at 8 weeks and log_2_ fold-change = 0.46, adj. *P*-value = 4.59E−4 at 16 weeks) “upstream” molecule particularly highlighted to contribute to the homing of lymphocytes through its effects on downstream DEGs was the innate immune signal transduction adaptor—myeloid differentiation primary response 88 (MYD88) (**Figure 9B**). Particularly, IL-18 binding and signaling can also activate MYD88 and induce downstream activation of NF-κB (40), and MYD88 is an important protein for TLR and IL1R-mediated signaling (41). Therefore, elevated levels and binding of IL-18 and activation of MYD88 are potentially key molecular mechanisms driving or enhancing the infiltration of lymphocytes into the affected chronically inflamed LGs. Camoteskimab is an anti-IL-18 monoclonal antibody (mAb) (42) (dotted square in **Figure 9A**) that is in clinical trials for the treatment of other inflammatory conditions such as atopic dermatitis (43).

## 4 Discussion

In the present study, RNA-seq was used to analyze the transcriptomic profiles of inflamed LGs from a Sjogren’s disease murine model and to compare them with the gene expression profiles of healthy LGs from a wild-type control group. Results from the differential gene expression analysis of diseased LGs at 3, 8, and 16 weeks versus age-matched control samples were uploaded to the QIAGEN IPA software and further analyzed to investigate the potential main molecular mechanisms and biological processes driving the development and overall pathophysiology of chronic LG inflammation in Sjogren-associated DED. Our RNA-seq datasets and their downstream biological analyses revealed the differential expression of several genes involved in progressively increasing the movement and trafficking of immune cells into the LG while also highlighting the up- or downregulation of signaling pathways involved in the modulation of immune responses and inflammation.

Based on our differential gene expression analyses, the most significantly altered pathways in the diseased LGs of NOR mice were primarily related to immune system processes involving cellular immune responses and cytokine-mediated signaling pathways. In particular, the cytokine storm signaling pathway in response to pathogens was the most significantly positively enriched canonical pathway identified in our analysis. The very strong positive enrichment of these pathways is supported and driven by the vast number of chemokines and cytokines that were found to be upregulated in diseased LGs when compared to healthy controls. Cytokine storm defines the phenomenon of significantly elevated levels of circulating chemokines, cytokines, and interferons and, as in SjD, is characterized by the overactivation of immune cells, which results in substantial inflammation and tissue damage (44, 45). In addition to the numerous immune cells that secrete chemokines and cytokines, endothelial and epithelial cells have also been described to produce such inflammatory stimuli. In particular, SG ductal epithelial cells can secrete chemokines, pro-inflammatory cytokines, and damage-associated molecular patterns (DAMPs), thus also contributing to the recruitment and activation of inflammatory cells (46).

Besides highly upregulated cytokine molecules in our datasets such as *Ifng* and *Tnf*, which were the most significantly predicted “upstream” regulators driving most of the transcriptomic changes observed in our differential expression datasets, multiple toll-like receptors (TLRs) upregulated in the diseased LGs when compared to healthy controls were also identified as significant molecular regulators driving the downstream differential expression. TLRs are pattern recognition receptors (PRRs) crucial for recognizing pathogen-associated molecular patterns (PAMPs) and DAMPs, inducing both innate and adaptive immune responses (29, 30, 44, 47). Upon recognition of DAMPs, TLRs activate downstream signaling pathways, leading to the release of chemokines, cytokines, interferons, and reactive oxygen species (44). In particular, *Tlr1*, *Tlr2*, *Tlr3*, *Tlr6*, *Tlr7*, *Tlr8*, *Tlr9*, *Tlr11*, *Tlr12*, and *Tlr13* were all upregulated in our 8- and/or 16-week pairwise comparisons. In addition to TLRs, other main PRRs including several c-type lectin receptors (*Clec2d*, *Clec7a*, *Clec9a*, and *Clec5a*, among others) and NOD-like receptors (*Nod1*, *Nod2*, *Nlrc4*, and *Nlrp3*, among others) were also significantly upregulated in the LGs of NOR mice compared to controls. The upregulation of these multiple PRRs therefore highlights the potential important role of DAMPs from damaged or dead cells, and even potential PAMPs as the result of bacterial or viral infections to the ocular surface, in activating the innate immune system and promoting the downstream expression of inflammatory mediators in autoimmune dacryoadenitis. Moreover, significantly elevated TLR7 expression levels have been reported in the peripheral blood mononuclear cells (PBMCs) and SGs of SjD patients (47, 48), with TLR7 and TLR9 expression detected in the ductal epithelial cells and lymphocytes of parotid SGs of SjD patients (47). These findings highlight the important role of TLRs in the development of SjD and position these PRRs as important mediators of the persistent immune and inflammatory responses occurring in the LGs and SGs of affected patients. Thus, the use of compounds that target specific TLRs, such as the ones listed by L. Alexopoulou (47), may provide a new therapeutic avenue for the treatment of autoimmune diseases and Sjogren-associated exocrine dysfunction.

Notably, the PPAR signaling pathway was among the few canonical pathways predicted to be inactivated or downregulated in our comparison analysis. As nuclear receptors and ligand-dependent transcription factors, PPARs can influence the transcription patterns of downstream genes involved in regulating several processes such as energy production, lipid metabolism, and inflammation (49). There are three PPAR isoforms (PPARA, PPARG, and PPARD; alpha, gamma, and delta, respectively) that are each encoded by a separate gene (50). In our datasets, the gene encoding the alpha isoform (*Ppara*) was downregulated, while *Ppard* (encoding the delta isoform) was upregulated in the diseased LGs. Consistent with our results, the downregulated gene expression of PPARA was also observed in the LGs of NOD.H2^b^ mice (another mouse model of SjD-associated LG inflammation) when compared to control samples (32). PPARA activation has been reported to inhibit NF-κB signaling, precisely by inhibiting the acetylation of p65 NF-κB, thus reducing the production of pro-inflammatory cytokines (49, 51). Furthermore, PPARG, which has been found to exert anti-inflammatory effects in epithelial cells and disease animal models, was previously described to be reduced in the SG ductal epithelia of SjD patients, minimizing its anti-inflammatory functions on constitutively and intrinsically activated NF-κB and IL-1β pathways (27, 28). Importantly, the *in vitro* treatment of SG epithelial cells with PPARG agonists significantly reduced NF-κB and cell apoptosis that was induced by pro-inflammatory agents (28). In the context of Sjogren-associated LG chronic inflammation and secretory dysfunction, treatment with fenofibrate, a PPARA agonist and FDA-approved hypolipidemic drug (32), was previously shown to reduce lymphocytic infiltrates in the LG, increase tear secretion, and improve corneal surface health by modulating several Th cell and T regulatory cell responses in the LGs of NOD mice (26). These findings corroborate the promising therapeutic potential of PPAR activation as a strategy for the treatment of chronic LG inflammation and secretory hypofunction.

In addition to significantly impacting the migration/recruitment and activation of lymphocytes and overall promoting persistent immune and inflammatory responses, the transcriptomic changes in the diseased LGs also pointed to potentially altered biological processes involving lipid metabolism and polyunsaturated fatty acid synthesis. In accordance with our results, Wu and co-authors reported the accumulation of cholesteryl esters in the LG of male NOD and identified an altered gene signature involved in lipid efflux and homeostasis, suggesting that such lipid deposition may contribute to the development and/or progression of LG inflammation in this SjD murine model (34). Such deposition of fat was clinically observed to be elevated in the LGs of SjD patients analyzed using magnetic resonance imaging and fat saturation sequencing techniques (52), indicating that lipid accumulation and altered lipid homeostasis are not only characteristic features present in LGs of SjD mouse models derived from the non-obese diabetic mouse strain, but also potential features of the human disease. The effects of this altered lipid metabolism and overall increased deposition and accumulation of lipids or fats in the normal LG physiology were previously studied by Xin He and colleagues (53). In their study, using a high-fat diet mouse model, it was demonstrated that hyperlipidemia resulted in a significant reduction of tear secretion, lipid droplet accumulation within the acinar cells of the LG, disruption of fatty acid oxidation, and elevated levels of immune cell infiltration as well as pro-inflammatory cytokines in the affected glands (53). Therefore, these findings suggest that altered lipid metabolism and improper lipid and/or fatty acid clearance could be a characteristic feature of Sjogren-associated LG dysfunction.

Aging is another major factor that contributes to the onset of DED by promoting LG alterations that result in decreased tear secretion and/or quality and increased glandular lymphocytic infiltration and periductal fibrosis (54, 55). Similar to the transcriptomic changes identified in our analysis, extensive changes in lipid homeostasis and lipogenesis have been recently reported in aged LGs from an aging mouse model that spontaneously develops DED (55). Particularly, genes encoding molecules involved in the regulation of lipid metabolism and lipid droplet formation, such as *Nr1h3* (Nuclear Receptor Subfamily 1 Group H Member 3) and *Apoe* (Apolipoprotein E), were also found to be upregulated in very aged (24 months) LGs from C57BL/6J (“black 6”) mice when compared to LGs from young (2-month-old) animals (55). Additional genes associated with lipid metabolism, fatty acid synthesis, or lipid droplet formation that were commonly significantly dysregulated in the LGs of our SjD mouse model and in aged LGs from “black 6” animals included downregulated genes such as *Erlin1*, *Acacb*, and *Mlxipl* and upregulated genes such as *Abcg1*, *Elovl5*, *Lipa*, *Ptafr*, *Cyp1b1*, and *Plin1*. The common differential expression of these genes involved in the regulation of lipid metabolism in distinctive DED animal models induced by autoimmune LG inflammation or organ dysfunction because of aging further emphasizes the potential implications that increased lipid accumulation and disrupted lipid metabolic processes may have in altering LG homeostasis and normal function, regardless of the degree of glandular inflammation. Further studies are necessary to elucidate the mechanisms driving lipid accumulation in the LGs of DED patients and animal models and understand how these lipid metabolic imbalances may disrupt normal LG epithelial functions.

One of the main molecular mechanistic networks identified in our analysis of the transcriptomic changes occurring in the diseased LGs of NOR mice when compared to control samples involved the upregulation/activation of *Il18* as a “master” upstream regulator of downstream DEGs that have been reported in the literature (based on the curated IPA Knowledgebase) to increase or activate the cellular activities of lymphocytes and the migration of other immune cells such as dendritic cells—which have been shown to infiltrate the SG of NOD mice before any lymphocytic infiltration, and also be present in the minor SGs of SjD patients at varying levels depending on the focal lymphocytic infiltrates also present (56–58). IL-18 is a member of the IL-1 family of cytokines with potent pro-inflammatory activities that is implicated in several autoimmune/inflammatory conditions such as rheumatoid arthritis, systemic lupus erythematosus, and inflammatory bowel disease (59). Consistent with the increased cellular activities of lymphocytes highlighted in our mechanistic network, IL-18 can strongly induce IFNG production and activate Th1 cells, CD8^+^ T cells, and natural killer cells, leading to enhanced type 1 immune responses (60, 61). In the context of DED, IL-18 protein levels were found to be significantly elevated in tears of both SjD-induced dry eye patients and age-related dry eye mice (62, 63). Additionally, both IL-18 mRNA and protein levels were significantly higher in conjunctival impression cytology-derived superficial epithelial cells of both SjD-positive and SjD-negative dry eye patients (63), strongly suggesting that this pro-inflammatory cytokine might be implicated in the development of LG chronic inflammation and DED. Thus, targeting IL-18 with either the anti-IL-18 mAb presented in this article or with other IL-18 inhibitors could offer a therapeutic approach to ameliorate aqueous-deficient DED induced by overactive lymphocytic activity and chronic glandular inflammation.

Potential limitations of the study include the fact that the differential expression of the genes reported in the present study and the activated or inhibited biological processes described as a result of such differential gene expression were not validated using additional molecular assays such as quantitative polymerase chain reaction (qPCR). To mitigate this limitation, we compared our differential gene expression results to other previously published and publicly available datasets from RNA-seq-based transcriptomic studies comparing diseased versus healthy LGs from other animal models of Sjogren-associated DED and differential expression data from parotid SGs of SjD-positive versus SjD-negative sicca human patients. However, the differential gene expression results presented in this study need to be validated at the protein level or with further functional assays before drawing more definitive conclusions. Additionally, and as with any transcriptomic or proteomic study, absolute conclusions of causative links or correlations between the identified DEGs and, in this case, Sjogren-associated DED, cannot be fully made. Thus, future studies are required to draw conclusive correlations between the candidate molecular biomarkers and the disease.

In conclusion, the RNA-seq-based transcriptomic analysis presented in this study revealed significant gene expression alterations taking place in chronically inflamed LGs from a well-established animal model of dry eye and human primary SjD, when compared to healthy controls. Besides playing critical roles in the recruitment and active migration of leukocytes into the LG interstitial, many of the DEGs were associated with the biosynthesis and metabolism of lipids, suggesting that in addition to inflammatory agents, an imbalanced lipid homeostasis also contributes to the autoinflammatory profile and exocrine dysfunction observed in the LGs of SjD patients and animal models. The present study highlights specific biological pathways/processes and key molecules that could be pharmacologically targeted to ameliorate LG chronic inflammation and hypofunction. However, further studies are needed to validate the transcriptomic changes reported in the present study at a functional level before examining the potential translational and clinical significance of targeting the underlying molecules and canonical pathways.

## Data Availability

The datasets presented in this study can be found in online repositories. The names of the repository/repositories and accession number(s) can be found below: https://www.ncbi.nlm.nih.gov/geo/, GSE309371.
