# Supplementary material for: Transcriptomic changes in the lacrimal glands of a Sjogren’s disease animal model highlight key molecular mediators and altered biological functions underpinning glandular inflammation and hypofunction
Source: Front Ophthalmol (Lausanne). 2026 Mar 13;6:1697924. doi: 10.3389/fopht.2026.1697924 (PMC13021454; doi:10.3389/fopht.2026.1697924)
Supplement: Supplementary file 1 [file Presentation1.pdf]

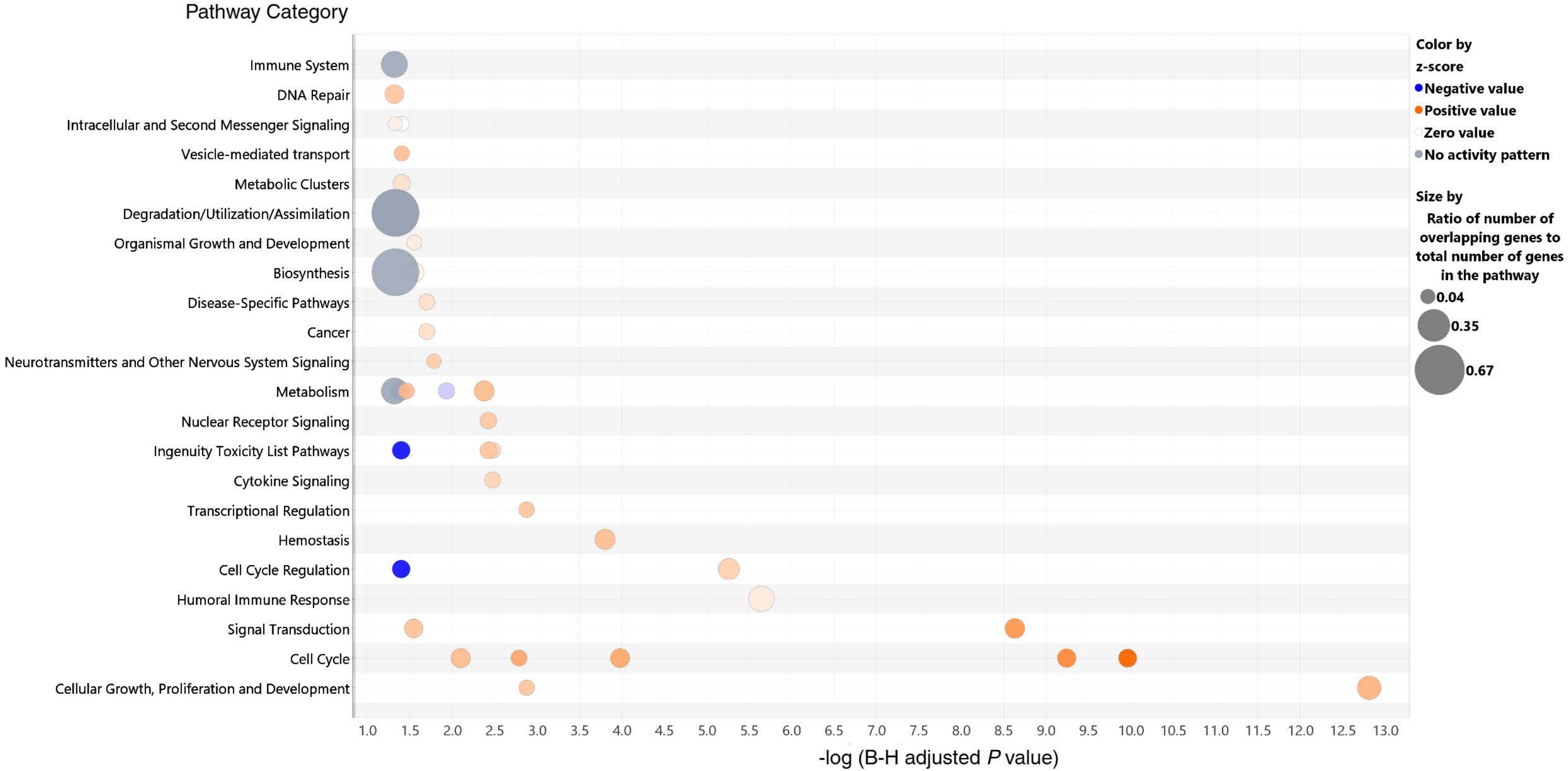

Supplementary Figure S1. Bubble plot of significantly enriched (-log Benjamini-Hochberg, BH adjusted *P* value ≥ 1.3) canonical pathways based on the differentially expressed genes identified in the 3-week NOR versus BALB/c comparison. Each bubble represents a canonical pathway belonging to a broader pathway category (shown on the y-axis). Pathways predicted to be activated/upregulated (positive z-score) are depicted by an orange bubble, while those pathways predicted to be inactivated/downregulated (negative z-score) are depicted by a blue bubble. Pathways for which an activity state cannot be predicted are represented by grey bubbles.

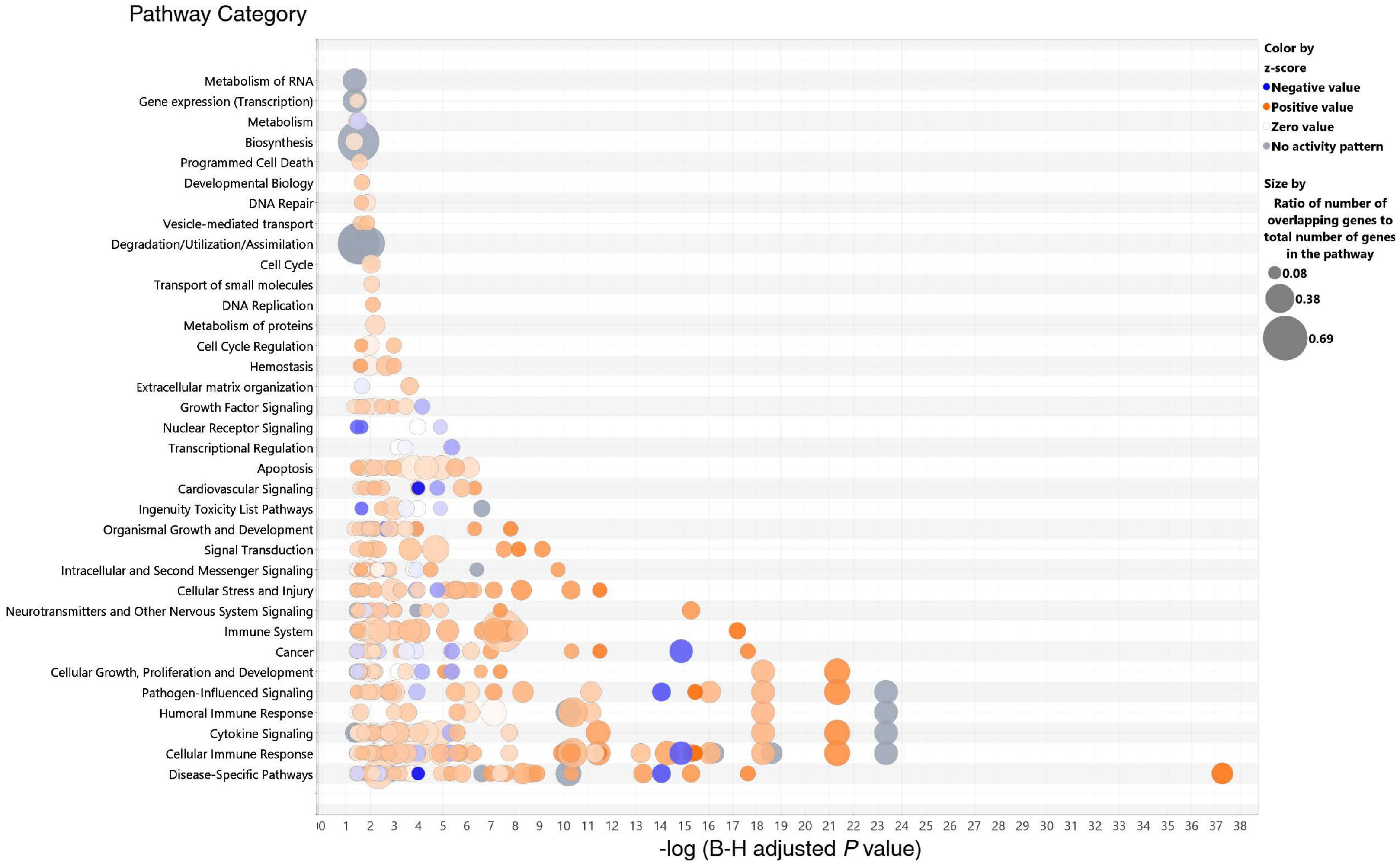

Supplementary Figure S2. Bubble plot of significantly enriched (-log Benjamini-Hochberg, BH adjusted *P* value ≥ 1.3) canonical pathways based on the differentially expressed genes identified in the 8-week NOR versus BALB/c comparison. Each bubble represents a canonical pathway belonging to a broader pathway category (shown on the y-axis). Pathways predicted to be activated/upregulated (positive z-score) are depicted by an orange bubble, while those pathways predicted to be inactivated/ downregulated (negative z-score) are depicted by a blue bubble. Pathways for which an activity state cannot be predicted are represented by grey bubbles.

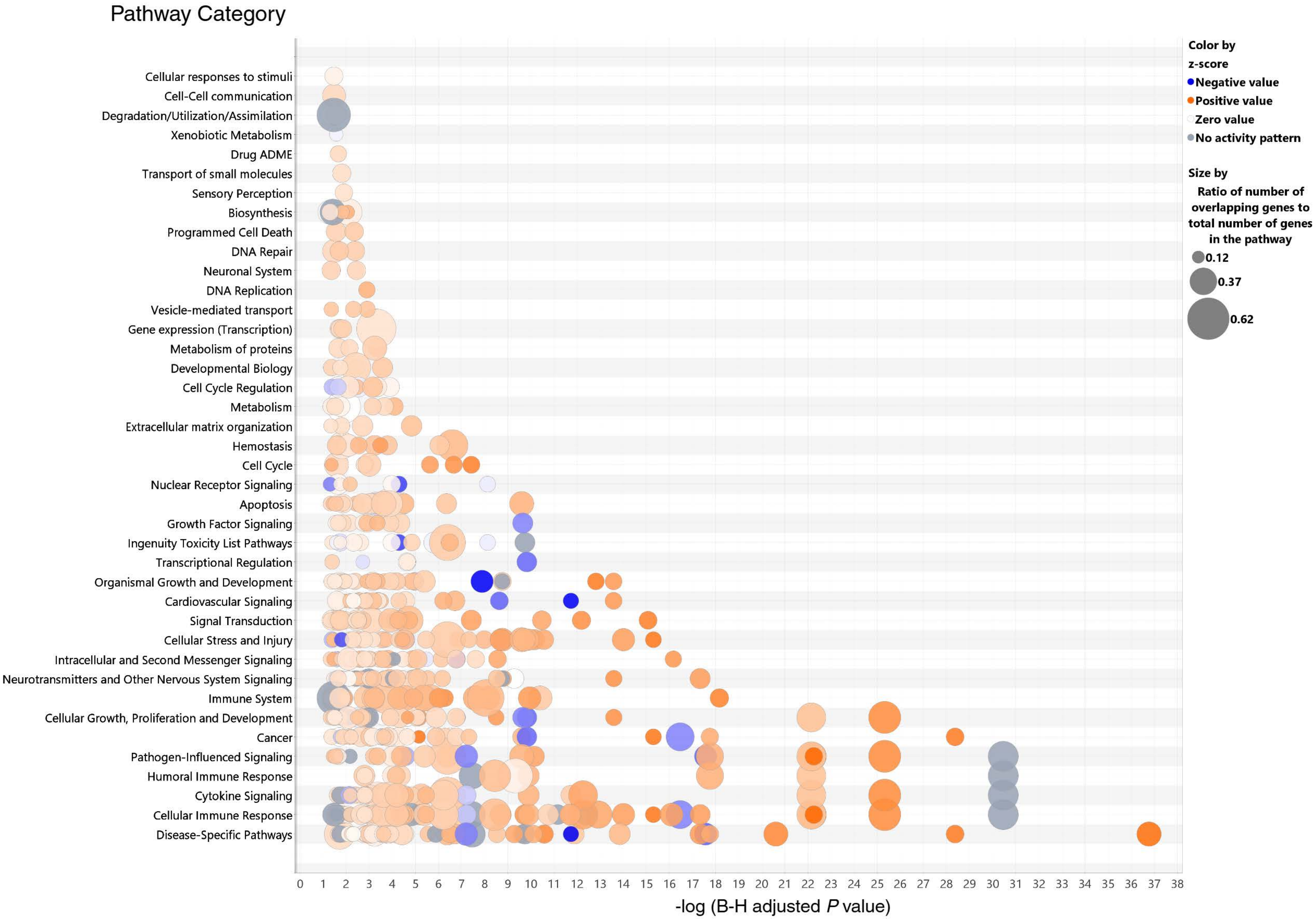

Supplementary Figure S3. Bubble plot of significantly enriched (-log Benjamini-Hochberg, BH adjusted *P* value ≥ 1.3) canonical pathways based on the differentially expressed genes identified in the 16-week NOR versus BALB/c comparison. Each bubble represents a canonical pathway belonging to a broader pathway category (shown on the y-axis). Pathways predicted to be activated/upregulated (positive z-score) are depicted by an orange bubble, while those pathways predicted to be inactivated/downregulated (negative z-score) are depicted by a blue bubble. Pathways for which an activity state cannot be predicted are represented by grey bubbles.

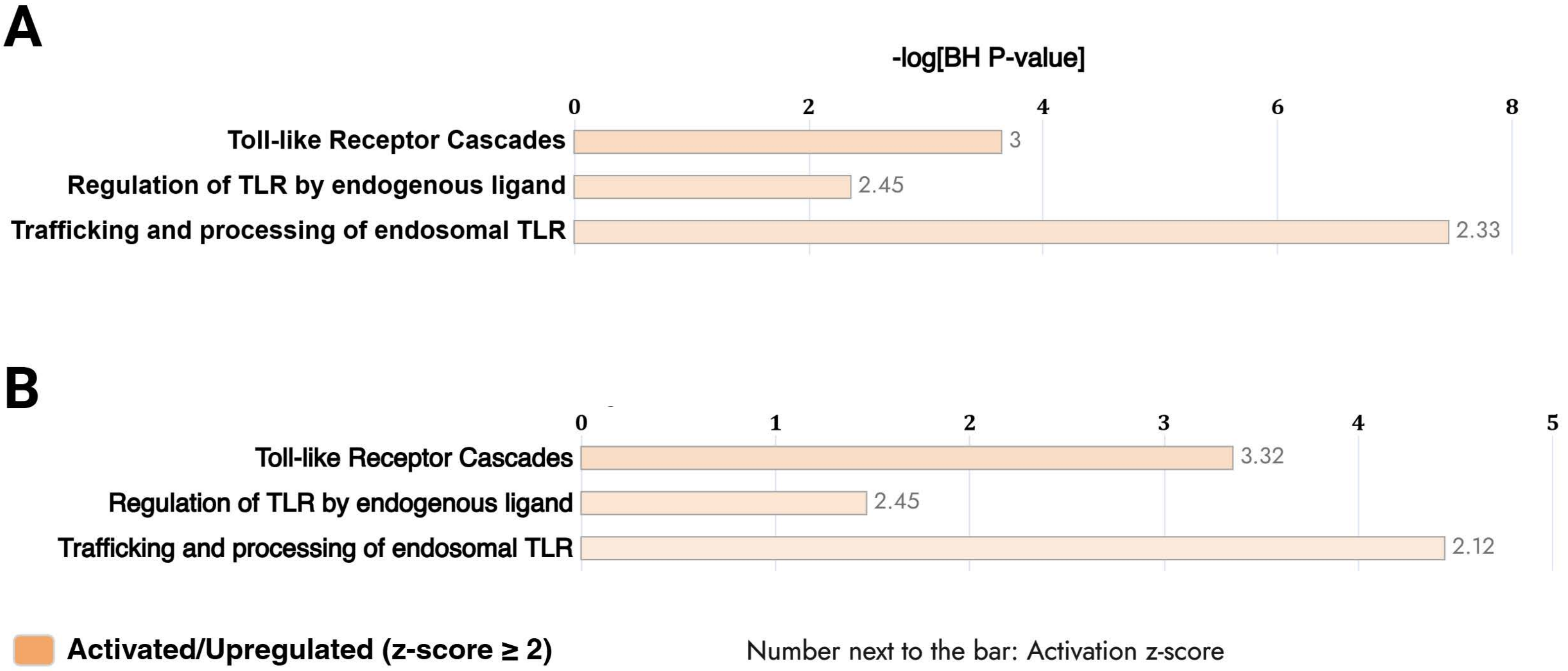

Supplementary Figure S4. Activation of Toll-like Receptor (TLR) related canonical pathways. (A) Bar plot showing TLR-related canonical pathways significantly predicted (Benjamini-Hochberg, BH adjusted *P* value ≤ 0.05) to be activated/upregulated in the lacrimal glands (LGs) of NOR mice at 8-weeks when compared to control samples. (**B**) Those same TLR-related pathways were also similarly activated/upregulated in the LGs of 16-week NOR mice compared to age-matched controls.

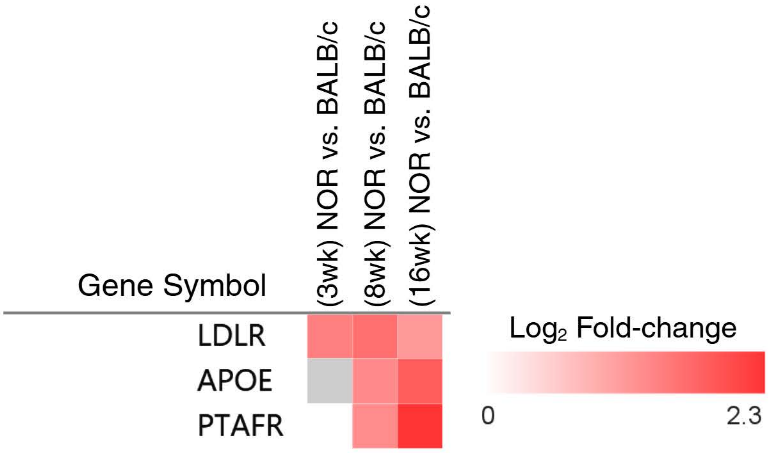

Supplementary Figure S5. Comparison Heatmap of the Temporal Differential Expression of Additional Genes related to Lipid Synthesis. Genes encoding additional molecules that participate in the transport of cholesterol or in lipid-mediated signaling processes that have been previously reported to be elevated in the LGs of other SjD murine models (31, 33), such as *Ldlr* (low-density lipoprotein receptor), *Apoe* (Apolipoprotein E), and *Ptafr* (platelet-activating factor receptor), were also significantly upregulated (depicted by red-colored heatmap squares) in our differential expression datasets. *Ptafr* was not differentially expressed in our 3-week NOR versus BALB/c comparison, hence, the empty or white heatmap square. *Apoe* was differentially expressed at 3-weeks, but below the set fold-change cutoff (absolute $\log_2$ fold-change ≥ 1), as indicated by the grey-colored heatmap square.

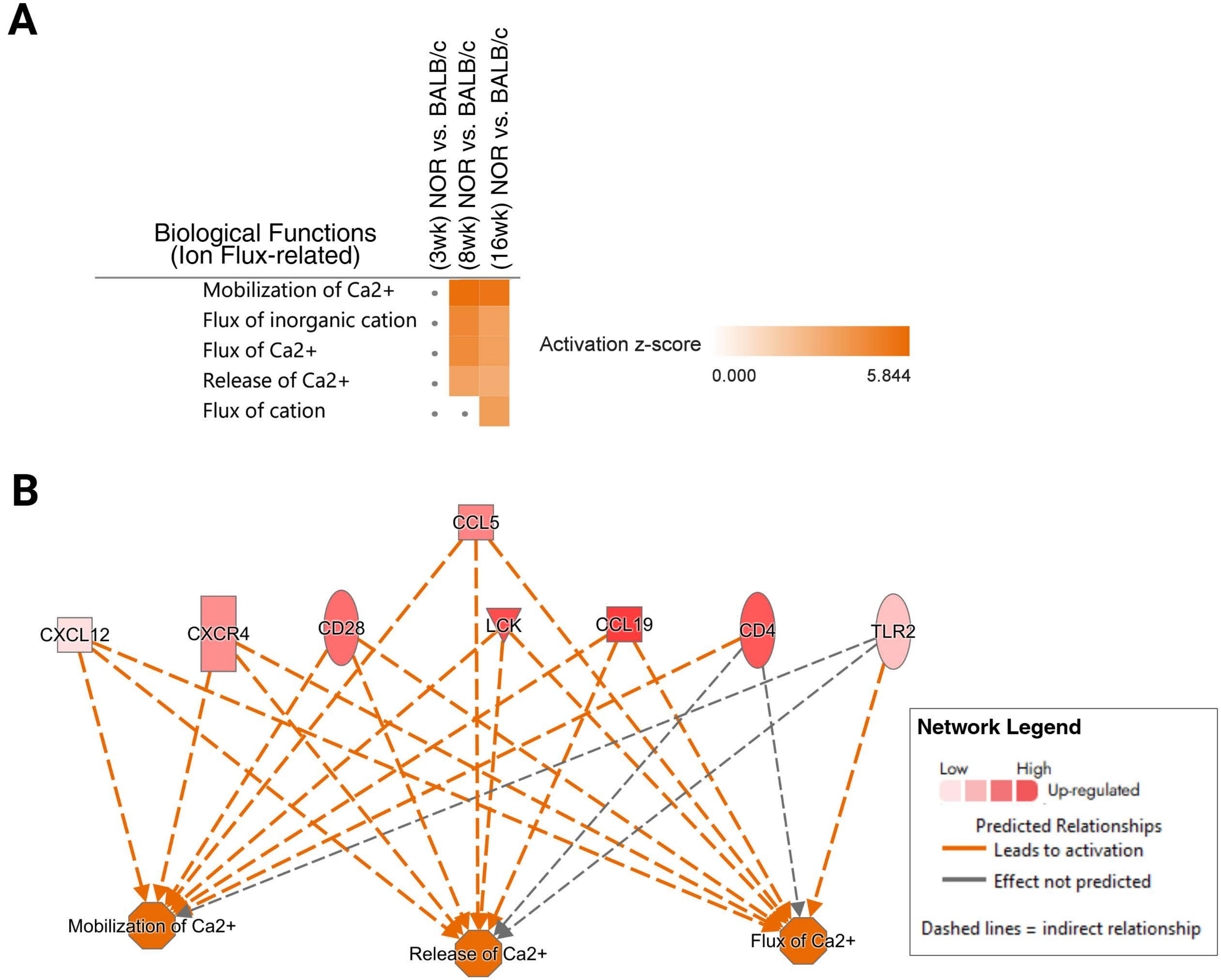

Supplementary Figure S6. Enhanced Mobilization/Flux of Calcium Ions. (**A**) Heatmap comparing the activation/ upregulation (z-score ≥ 2, orange-colored heatmap squares) of several biological processes related to the mobilization, flux, or release of calcium ions ($Ca2^{+}$) across our three pairwise comparisons. Shown biological functions (heatmap rows) not significantly activated at a particular time point (heatmap columns) are marked with a 'dot'. (**B**) Molecular mechanistic network depicting how the upregulation of dataset DEGs can increase the mobilization, release, and flux of $Ca2^{+}$.
